# Preventing High Fat Diet-induced Obesity and Improving Insulin Sensitivity through Neuregulin 4 Gene Transfer

**DOI:** 10.1038/srep26242

**Published:** 2016-05-17

**Authors:** Yongjie Ma, Mingming Gao, Dexi Liu

**Affiliations:** 1 Department of Pharmaceutical and Biomedical Sciences, University of Georgia College of Pharmacy, Athens, Georgia, United States of America

## Abstract

Neuregulin 4 (NRG4), an epidermal growth factor-like signaling molecule, plays an important role in cell-to-cell communication during tissue development. Its function to regulate energy metabolism has recently been reported. This current study was designed to assess the preventive and therapeutic effects of NRG4 overexpression on high fat diet (HFD)-induced obesity. Using the hydrodynamic gene transfer method, we demonstrate that *Nrg4* gene transfer in mice suppressed the development of diet-induced obesity, but did not affect pre-existing adiposity and body weight in obese mice. *Nrg4* gene transfer curbed HFD-induced hepatic steatosis by inhibiting lipogenesis and PPARγ-mediated lipid storage. Concurrently, overexpression of NRG4 reduced chronic inflammation in both preventive and treatment studies, evidenced by lower mRNA levels of macrophage marker genes including *F4/80, Cd68, Cd11b, Cd11c*, and macrophage chemokine *Mcp1*, resulting in improved insulin sensitivity. Collectively, these results demonstrate that overexpression of the *Nrg4* gene by hydrodynamic gene delivery prevents HFD-induced weight gain and fatty liver, alleviates obesity-induced chronic inflammation and insulin resistance, and supports the health benefits of NRG4 in managing obesity and obesity-associated metabolic disorders.

Neuregulin 4 (NRG4) is a member of the neuregulin family (NRG1–NRG4) that shares a common structure of epidermal growth factor (EGF)-like domains^1^. Similar to other neuregulin members, NRG4 activates type-1 growth factor receptors (ErbB3 and ErbB4 receptor) to initiate cell-to-cell signaling through tyrosine phosphorylation^1^,^2^. NRG4 was originally detected in the adult pancreas and muscle, and is considered the essential factor for tissue development^1^. Further studies demonstrate that NRG4 is present in human breast milk and developing intestine tissue, promoting epithelial cell survival and protecting against experimental necrotizing enterocolitis^3^,^4^. In addition, NRG4 has been found in prostate, breast and gastric cancers^5^,^6^,^7^.

More recent work by Rosell *et al*. showed that NRG4 is enriched in brown adipose tissue (BAT) and its expression is upregulated in cold-induced beige/brite cells^8^. *In vitro* studies demonstrated that NRG4-contained medium from differentiated brown adipocytes promotes neurite outgrowth^8^. Using NRG4-deficient mice, Wang *et al*. revealed that NRG4 is capable of attenuating hepatic lipogenic signaling and maintaining metabolic homeostasis^9^. The findings from these previous studies indicate that NRG4 may work as a novel adipokine with a possible role in maintaining energy and metabolic homeostasis.

In the current study, we used a gene transfer system to enhance NRG4 expression and investigated its potential to prevent and to provide therapeutic benefits to animals with diet-induced obesity and metabolic disorders. Our results show that an increase of NRG4 expression inhibits diet-induced chronic inflammation, improves insulin resistance, and prevents weight gain in a diet-induced animal model.

## Results

### NRG4 gene transfer prevents diet-induced weight gain

**Figure 1:**
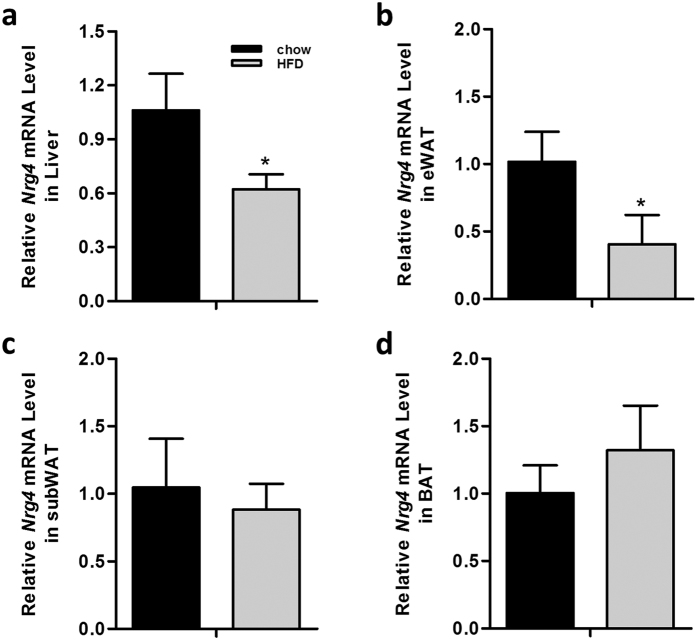
*Nrg4* gene expression was reduced in liver and EWAT of obese mice. Eight-week-old male C57BL/6 mice were fed a chow or HFD for total 12 weeks, Total RNA was extracted and relative mRNA levels of *Nrg4* was determined in liver (**a**), EWAT (**b**), SubWAT (**c**), and BAT (**d**) by real-time PCR. *P < *0.05* compared to chow-fed mice (n = 3).

**Figure 2:**
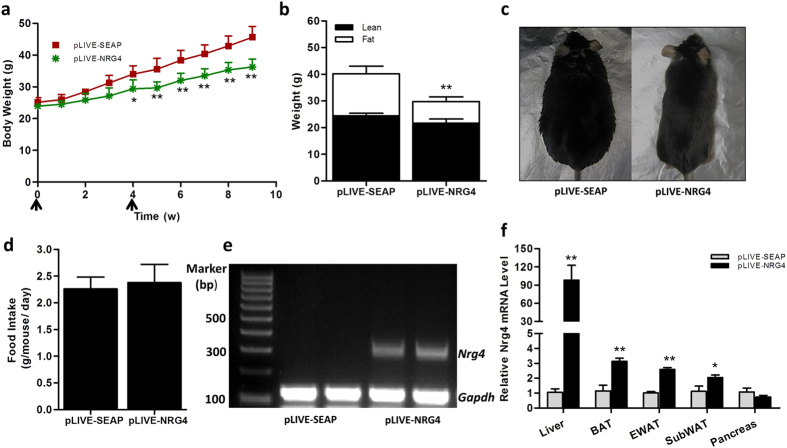
*Nrg4* gene transfer prevented high fat diet-induced weight gain. Eight-week-old C57BL/6 male mice were hydrodynamically injected via tail vein of 20 μg of pLIVE-NRG4 or pLIVE-SEAP control plasmid DNA (on days 1 and 28) and fed an HFD for 9 weeks. At the end of the experiment, total RNA was extracted from liver, EWAT, SubWAT, BAT, pancreatic tissues, and the relative mRNA levels of *Nrg4* gene were determined by real-time or regular PCR. (**a**) Body weight-time curve; (**b**) Fat mass and lean mass at the end of 9-week feeding; (**c**) Representative images of mice at the end of the experiment; (**d**) Average food intake; (**e**) Detection of *Nrg4* gene in the liver by regular PCR; and (**f**) *Nrg4* mRNA level in different tissues determined by real time PCR. Each data point represents the mean ± SD of 5 animals. *P < 0.05, **P < 0.01 compared to control animals injected with pLIVE-SEAP plasmid DNA.

First, we confirmed the mRNA levels of *Nrg4* in normal-weight and obese mice. Results in Fig. 1 show a reduction of the *Nrg4* transcript level in the liver and epididymal white adipose tissue (eWAT) of the obese mice by ~40% and ~60%, respectively, compared to age-matched chow-fed mice. There was no difference in the *Nrg4* transcript level in subcutaneous white adipose tissue (SubWAT) and BAT between normal-weight and obese mice. Results in Fig. 2a showed that hydrodynamic injections of pLIVE-NRG4 plasmids (on day 1 and day 28) were sufficient to protect mice from HFD-induced weight gain. After nine weeks of HFD feeding, pLIVE-NRG4-injected animals had an average body weight of 36 grams, 10 grams less than that of the control animals (~46 g) injected with control plasmid pLIVE-SEAP carrying the secreted embryonic alkaline phosphatase gene. The difference in body size was visually differentiable (Fig. 2c). Analysis of body composition revealed that the difference in body weight was primarily from fat mass (~49% reduction), not from lean mass (Fig. 2b). The average food intake between the two groups of animals was similar (Fig. 2d).

To test the effect of NRG4 increase on diet-induced obesity, we overexpress NRG4 by performing hydrodynamic gene delivery. Hydrodynamic-based gene transfer is an effective gene delivery method, which has been widely used for gene expression and functional analysis in whole animals^10^,^11^,^12^,^13^,^14^. Expression of NRG4 in animals receiving pLIVE-NRG4 plasmid transfer was confirmed by real-time PCR (RT-PCR) analysis and the results revealed that the mRNA level in the liver of the treated animals at the end of the experiment was approximately 97-fold higher than that of the control animals receiving two injections of pLIVE-SEAP control plasmids (Fig. 2f, Fig. S1). A high level of *Nrg4* gene transfer was also confirmed by regular PCR analysis (Fig. 2e). In addition, an approximate 2–3-fold increase of *Nrg4* expression was detected in the adipose tissues including BAT, eWAT, and SubWAT, but not in the pancreas (Fig. 2f). These results prove that NRG4 overexpression was responsible for blocking high fat diet-induced weight gain.

### NRG4 suppresses chronic inflammation in eWAT and stimulates BAT thermogenesis

**Figure 3:**
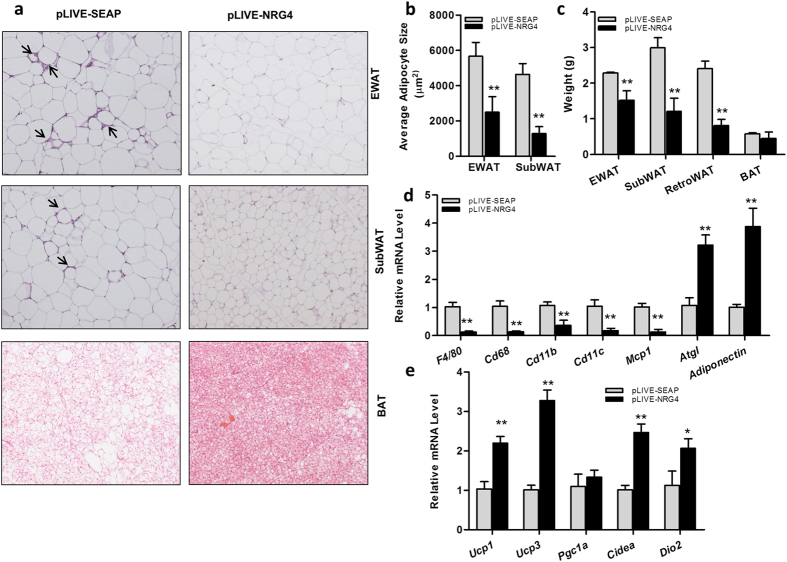
NRG4 suppressed chronic inflammation in EWAT and stimulated expression of thermogenic genes in BAT. Mice were sacrificed after 9 weeks of HFD feeding. Fat pads including EWAT, SubWAT and BAT were collected and weighed. Adipose tissues were fixed in 10% neutrally buffered formalin and H&E staining was performed. Total RNA was extracted from EWAT and BAT and relative mRNA levels of selected genes were determined by real-time PCR. (**a**) Representative images of H&E staining of EWAT, SubWAT and BAT (100×); (**b**) Average size of adipocytes in EWAT and SubWAT (calculated from measurements of 200 adipocytes from 5 separate slides); (**c**) Weight of different adipose pads; (**d**) Relative mRNA levels of selected macrophage marker genes including *F4/80, Cd68, Cd11c*, chemotactic factor gene *Mcp1, Adiponectin* and *Atgl;* (**e**) Relative mRNA levels of thermogenic genes including *Ucp1, Ucp3, Pgc1α, Cidea* and *Dio2.* *P < 0.05, **P < 0.01 compared to that of control animals injected with pLIVE-SEAP (n = 5).

Consistent with the lower fat mass in pLIVE-NRG4-injected animals, H&E staining of adipocytes (Fig. 3a) and further quantitative analysis (Fig. 3b) revealed that *Nrg4* gene transfer blocked the hypertrophy of adipocytes in eWAT and SubWAT by more than 50%, which was in line with the weight difference in the fat pads (Fig. 3c). Crown-like structures that are seen in the adipose tissue sections of the control animals are not detected in mice with the hydrodynamic delivery of pLIVE-NRG4 plasmids, indicating that NRG4 overexpression is capable of preventing diet-induced macrophage infiltration. Results from RT-PCR analysis (Fig. 3d) show that *Nrg4* gene transfer suppressed HFD-induced expression of macrophage-specific marker genes including *F4/80, Cd68, Cd11b* and *Cd11c* by ~88%, ~87%, ~64% and ~83%, respectively. *Nrg4* gene transfer also reduced monocyte chemotactic protein 1 (*Mcp1*) expression, a chemokine gene that regulates migration and infiltration of macrophages. Similarly, *Nrg4* gene transfer enhanced M2 macrophage marker gene *Cd163* (Supplemental Fig. S2). These results demonstrate the anti-inflammatory effect of NRG4 overexpression on diet-induced obesity. *Nrg4* gene transfer also increased the expression of adiponectin (~3.2-fold) and adipose triglyceride lipase (*Atgl*, ~3.8-fold) genes.

Results in Fig. 3a (lowest panel) show fewer lipid droplets in BAT of pLIVE-NRG4-injected mice than those of the control animals, as determined by the density of vacuole-type structures. BAT is known as the thermogenic organ, facilitating utilization of extra glucose or lipids to generate heat^15^,^16^. To evaluate the effect of NRG4 overexpression on body temperature, we measured the rectal temperature of the animals with a digital thermometer. The results in Supplemental Fig. S1 showed that pLIVE-NRG4-injected mice had an increase of ~1.0 °C body temperature one day after the gene transfer and maintained this relatively higher body temperature for two weeks. Moreover, NRG4 overexpression also enhanced the expression of BAT thermogenic genes, including uncoupling protein *Ucp1* (~ 2.2-fold), cell death-inducing DNA fragmentation factor-α-like effector A (*Cidea*, ~2.4-fold), and the type 2 deiodinase (*Dio2,* ~2.0-fold) (Fig. 3e), suggesting that *Nrg4* gene transfer influences energy expenditure in BAT. Similar effects of *Nrg4* gene transfer on browning marker genes were also seen in the inguinal WAT (Supplemental Fig. S4).

### *Nrg4* gene transfer prevents diet-induced hyperinsulinemia and insulin resistance

**Figure 4:**
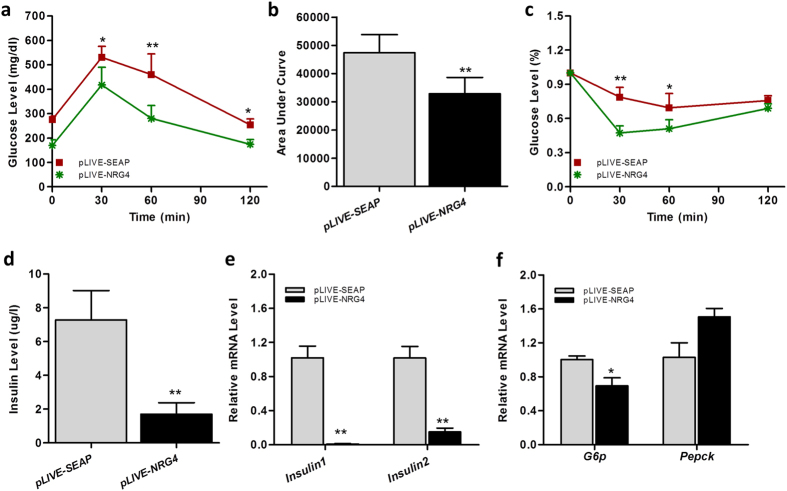
*Nrg4* gene transfer improved hyperinsulinemia and insulin resistance of animals fed an HFD. (**a**) Time-dependent blood glucose level followed by IP injection of glucose (1.5 g/kg); (**b**) Area under the curve from glucose tolerance test (**a**); (**c**) Relative ratio of glucose concentration upon IP injection of insulin (0.75 U/kg); (**d**) Serum insulin levels at the end of the 9-week HFD feeding; (**e**) Relative mRNA levels of *Insulin1* and *Insulin2* in pancreas; (**f**) mRNA levels of *G6p* and *Pepck* gene involved in glucogenesis. Each data point represents the mean ± SD of 5 animals. *P < 0.05, **P < 0.01 compared to control animals.

To assess the impact of *Nrg4* gene transfer on insulin sensitivity and glucose homeostasis, glucose tolerance tests were performed and the results in Fig. 4a,b revealed a lower peak level and much higher clearance rate of intraperitoneally injected glucose in animals with *Nrg4* gene transfer. pLIVE-NRG4-injected mice were more sensitive to insulin administration than the control mice (Fig. 4c). *Nrg4* gene transfer dramatically suppressed an HFD-induced increase in the blood insulin level (7.3 μg/L vs. 1.6 μg/L, Fig. 4d). Compared to the control animals, pancreatic mRNA levels of *Insulin1* and *Insulin2* genes were markedly lower in mice with *Nrg4* gene transfer (Fig. 4e). NRG4 overexpression also suppressed glucose 6-phosphate (*G6p*) gene expression, but not the phosphoenolpyruvate carboxykinase gene (*Pepck)* that is directly involved in glucogenesis (Fig. 4f). These results validate our hypothesis that NRG4 protects animals from obesity-associated insulin resistance.

### *Nrg4* gene transfer blocks hepatic lipogenesis and lipid storage

**Figure 5:**
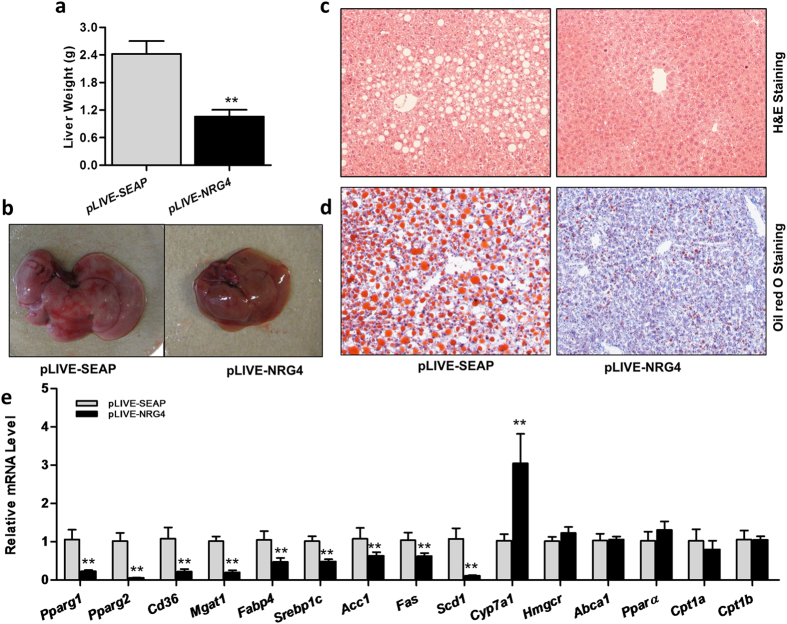
*Nrg4* gene transfer inhibited lipogenesis and lipid accumulation in mouse liver. At the end of the 9-week HFD feeding, mice were sacrificed and livers were collected and fixed for histochemistry. (**a**) Liver weight at the end of experiment; (**b**) Representative images of mouse livers; Liver sections were stained with H&E (**c**) or Oil Red O (**d**) (original magnifications 100×); (**e**) Relative mRNA levels of genes involved in hepatic lipid metabolism. Each data point represents the mean ± SD of 5 animals. *P < 0.05, **P < 0.01 compared to control animals.

Obesity is associated with fatty liver disease^17^. Results in Fig. 5a show that *Nrg4* gene transfer inhibits liver enlargement by 2.4-fold compared to that of the control animals (1.0 g *vs.* 2.4 g); a photograph of the liver discerned this outcome (Fig. 5b). H&E-stained liver sections showed a relatively normal liver structure in mice with *Nrg4* gene transfer, compared to the extensive hepatocyte vacuolation in control animals (Fig. 5c). Oil Red O staining of liver sections also confirmed the difference (Fig. 5d). These results provide direct evidence in support that NRG4 overexpression is capable of blocking hepatic lipid accumulation.

We performed RT-PCR analyses to assess the effect of *Nrg4* gene transfer on the expression of genes involved in hepatic lipid metabolism. Results in Fig. 5e show that NRG4 overexpression markedly suppressed the expression of lipogenic genes in the liver, including sterol regulatory element-binding protein 1c (*Srebp1c,* ~52%), acetyl-CoA carboxylase 1 (*Acc1,* ~37%), fatty acid synthase (*Fas,* ~38%), and stearoyl-CoA desaturase-1 (*Scd1,* ~89%). *Nrg4* gene transfer also reduced the expression of peroxisome proliferator-activated receptor gamma (*Pparγ1*, ~77%), *Pparγ2* (~99%), and their target genes, including *Cd36* (~88%), fatty acid binding protein 4 (*Fabp4*, ~53%), and monoacylglycerol O-acyltransferase 1 (*Mgat1*, ~80%). In regard to cholesterol metabolism, NRG4 overexpression enhanced cholesterol 7 α-hydroxylase (*Cyp7a1*) gene expression by ~3-fold, but there was no change of the mRNA level for 3-hydroxy-3-methylglutaryl coenzyme A reductase (*Hmgcr*) and the ATP-binding cassette transporter (*Abca1*) genes (Fig. 5e). At the end of the 9-week experiment, *Nrg4* gene transfer did not affect *Pparα* and its target gene, carnitine palmitoyl-transferase I (*Cpt1*). Collectively, these data demonstrated that prolonged *Nrg4* gene expression alleviates obesity-associated fatty liver.

### NRG4 overexpression improves insulin sensitivity in obese mice without changing body weight

**Figure 6:**
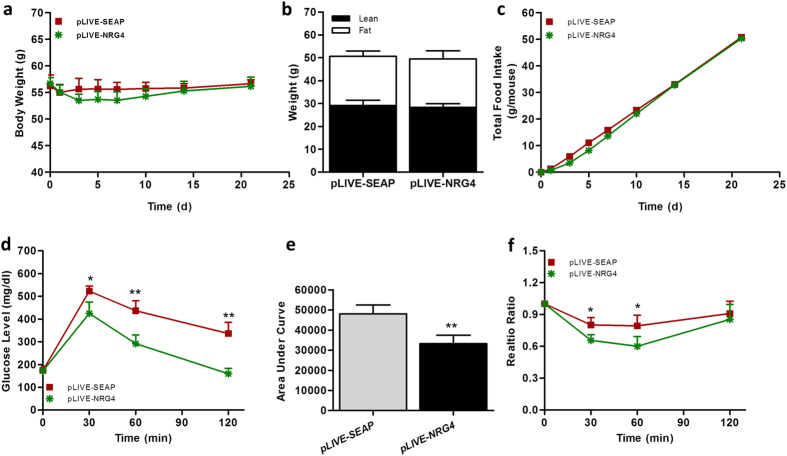
NRG4 improved insulin resistance in obese mice. C57BL/6 obese mice fed an HFD were hydrodynamically injected via tail vein with 20 μg of pLIVE-NRG4 or pLIVE-SEAP plasmid for 3 weeks. (**a**) Change of body weight; (**b**) Fat and lean mass measured 3 weeks after gene transfer; (**c**) Total food intake; (**d**) Time-dependent blood glucose level in glucose tolerance test (1.5 g/kg); (**e**) Area under the curve from glucose tolerance test; (**f**) Relative ratio of glucose concentration in insulin tolerance test (0.75 U/kg). *P < 0.05, **P < 0.01 compared to control animals (*n* = 5).

The preventive effect of NRG4 on HFD-induced obesity has been clearly demonstrated. Next, we assessed the effects of *Nrg4* gene transfer on obese mice. Diet-induced obese mice (average body weight 56 g) were randomly divided into two groups and injected with pLIVE-NRG4 or pLIVE-SEAP plasmids using a hydrodynamics-based procedure. Results in Fig. 6a–c show that *Nrg4* gene transfer failed to reduce body weight and did not change fat or lean mass. There was no difference in food intake between mice receiving pLIVE-NRG4 or pLIVE-SEAP plasmid DNA. However, NRG4-treated mice exhibited a much higher clearance rate of glucose in the glucose tolerance test (GTT) assay (Fig. 6d), which was better represented by the calculated area under the curve (AUC, Fig. 6e). Improvement of insulin sensitivity in obese mice was also seen in an insulin tolerance test. Taken together, these data demonstrate that *Nrg4* gene transfer improves insulin sensitivity in obese mice.

### NRG4 overexpression reduced chronic inflammation in obese mice

**Figure 7:**
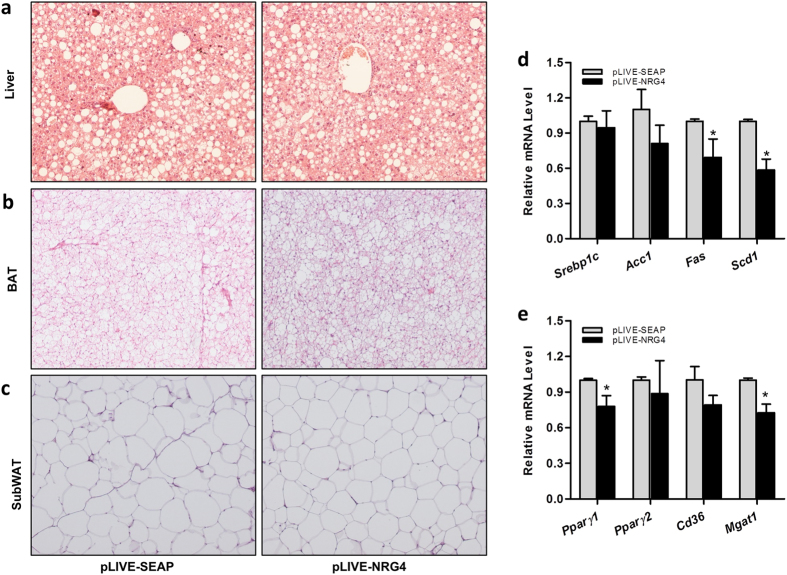
Effects of NRG4 overexpression on lipid storage in obese mice. (**a–c**) Representative images of H&E staining of the liver, EWAT and SubWAT (original magnifications 100×); (**d**) mRNA levels of genes involved in lipogenesis in the liver; (**e**) mRNA levels of genes involved in hepatic lipid accumulation. *P < 0.05, **P < 0.01 compared to control animals (n = 5).

H&E staining was performed and the results in Fig. 7a–c show no structural change of the liver, BAT, and SubWAT among the treated or control animals. Large lipid droplets in the liver and adipocytes were found in both groups of animals, indicating that lipid storage in these tissues was not affected. Gene expression analysis showed that *Nrg4* gene transfer did not change the mRNA levels of *Srebp1c* and *Acc1,* but did reduce *Fas* and *Scd1* mRNA levels by ~30% and ~42%, respectively (Fig. 7d). In addition, NRG4 overexpression reduced mRNA levels of *Pparγ1* (~22%) and *Mgat1* (~28%), but not that of *Pparγ2* and *Cd36* (Fig. 7e).

**Figure 8:**
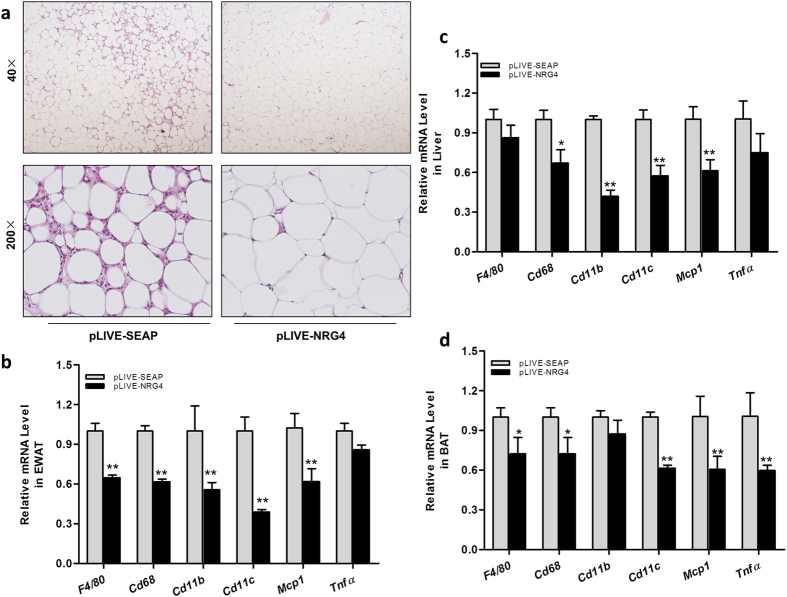
NRG4 overexpression reduced chronic inflammation in obese mice. At the end of the 3-week. after gene transfer, obese animals were sacrificed. (**a**) Images of EWAT stained with H&E; (**b–d**) Relative mRNA levels of macrophage marker genes in EWAT, liver, and BAT including *F4/80, Cd68, Cd11b, Cd11c* and inflammatory factor *Mcp1* and *Tnfα*. *P < 0.05, **P < 0.01 compared to control animals (n = 5).

Results in Fig. 8a show significant macrophage infiltration into EWAT of the obese animals, as reflected by multiple crown-like structures in H&E-stained EWAT sections. However, the crown-like structures were less obvious in animals with *Nrg4* gene transfer. RT-PCR was carried out to confirm the difference in the macrophage activation in the WAT of the treated and controlled animals. Results in Fig. 8b show a significant reduction in the mRNA levels of macrophage marker genes in mice with *Nrg4* gene transfer, including *F4/80, Cd68, Cd11b,* and *Cd11c* in EWAT of obese mice. In addition, the mRNA level of inflammatory factor gene *Mcp1* was also reduced by ~40%. A similar effect of *Nrg4* gene transfer on gene expression was detected in the liver and BAT of mice with NRG4 overexpression (Fig. 8c,d). These results suggest that NRG4 overexpression is capable of inhibiting obesity-related chronic inflammation.

## Discussion

In this study, we systematically examined the effects of *Nrg4* gene transfer on high fat diet-induced obesity and obesity-associated metabolic changes. The results show that *Nrg4* gene transfer protects mice from diet-induced weight gain and adiposity (Figs 2 and 3), and inhibits fatty liver and insulin resistance (Figs 4 and 5). The beneficial effects are correlated with an inhibition of lipogenesis, lipid storage, and chronic inflammation (Figs 3 and 5). NRG4 overexpression in obese mice did not affect pre-existing fat and body weight, but reduced chronic inflammation and improved obesity-related insulin resistance (Figs 6, 7, 8).

Obesity increases the risk of type 2 diabetes, cardiovascular diseases, and certain cancers, which are among the leading causes of death worldwide^18^. Although a cure for obesity is important, treatments are costly, and success so far is limited due to a lack of safe and effective methods. Prevention that is capable of benefiting a larger majority of the population and halting obesity-related metabolic disorders is more desirable and effective. Weight control programs through dietary intervention and enhanced physical activities have been popular in the past, but the successes have been limited to a small percentage of people since persistence in restricting food intake and maintaining regular exercise can be difficult to accomplish^19^. Evidently, efforts to explore new methods and strategies are urgently needed in dealing with the obesity epidemic.

NRG4 was originally identified as a factor that plays a critical role in tissue development^1^. However, a high level of expression in BAT and cold-induced expression in WAT suggest that NRG4 may act as an adipokine with a distinct function^8^. Since BAT plays an essential role in regulating body temperature and thermogenesis^15^,^16^, as well as maintaining systemic lipid and glucose homeostasis^20^,^21^,^22^, an elevated level of NRG4 in BAT indicates that NRG4 may be involved in energy metabolism and maintenance of metabolic homeostasis. A lower mRNA level of *Nrg4* (Fig. 1) in EWAT and in the liver of diet-induced obese mice appears to support such a prediction. In addition, a decrease in NRG4 levels was also observed in patients with non-alcoholic fatty liver disease (NAFLD)^23^.

NRG4 significantly inhibited lipogenesis by suppressing *Srebp1c* and its target genes, which is consistent with the previous results obtained from NRG4-deficient mice^9^. Elevating SREBP1c expression promotes lipid accumulation in the liver and fat tissue^24^,^25^, and HFD feeding induces SREBP1c-mediated lipogenesis in obese mice^26^,^27^. However, inhibition of lipogenesis exerts a positive effect on metabolic diseases^28^,^29^. Therefore, suppression of lipogenesis by NRG4 plays, at least in part, an important role in preventing diet-induced obesity. In addition, *Nrg4* gene transfer inhibited expression of *Pparγ* and its target genes (Fig. 5e). PPARγ is a transcription factor regulating the expression of genes responsible for lipid storage and adipocyte differentiation^30^. Previous studies have shown that elevated expressions of *Pparγ, Cd36*, and *Mgat1* genes are directly linked to hepatic steatosis^31^,^32^,^33^. Therefore, by targeting the hepatic PPARγ pathway, NRG4 protects against obesity-induced hepatic steatosis.

Several studies have demonstrated the interaction of NRG4 with inflammation. NRG4 levels in animals with ulcerative colitis (UC) and Crohn’s disease are reduced compared to un-inflamed controls. NRG4 treatment blocked inflammatory cytokine-induced apoptosis in colon epithelial cells and protected animals from experimental inflammatory bowel disease^3^,^4^. This conclusion agrees with our observation that NRG4 significantly reduced crown-like structures in EWAT and lowered expression of macrophage marker genes such as *F4/80, Cd68, Cd11,* and inflammatory factor *Mcp1* (Figs 3 and 8). These results also align with our previous reports that suppressing HFD-induced inflammation by either antioxidant^34^ or macrophage elimination^35^ is sufficient to block HFD-induced obesity. However, results shown in Fig. 6a,b suggest that blockage of inflammation is not sufficient to reduce pre-existing body weight and fat mass in obese mice, indicating further strategy is needed to mobilize stored fat.

Reduction of inflammation by NRG4 appears to benefit insulin sensitivity in both prevention and treatment studies. It is known that chronic low-grade tissue inflammation is a major detrimental factor for obesity-associated metabolic disorders such as insulin resistance. Long-term nutrient overload in adipose tissue and the liver increases a stress response, leading to the recruitment and activation of macrophages and thus the activation of inflammatory pathways^36^. Consequently, insulin action is blunted in the stressed tissues by inhibiting insulin-signaling transduction, causing cellular insulin resistance^37^,^38^. Therefore, inhibiting inflammatory and macrophage infiltration by NRG4 contributes to improved insulin sensitivity in both the prevention and treatment studies. This conclusion is in agreement with the results of the studies where normal weight rats and *db/db* mice were used to assess whether acute injection of NRG1 protein improves glucose tolerance through the activation of ErbB3 receptors and phosphorylation of protein kinase B (PKB) and forkhead box protein O1(FOXO1) in the liver^39^,^40^.

In conclusion, the current study demonstrates that *Nrg4* gene transfer inhibits lipogenesis and reduces chronic inflammation, resulting in the prevention of HFD-induced adiposity and fatty liver, and the improvement of insulin sensitivity. These findings provide direct evidence in support of the potential health benefits of NRG4 in managing obesity and obesity-associated metabolic disorders.

## Materials and Methods

### Materials

C57BL/6 mice were purchased from Charles River (Wilmington, MA). High-fat diet was obtained from Bio-serv (Frenchtown, NJ) (catalog number: F3282, 60% kJ/fat). The pLIVE and pLIVE-SEAP plasmids were purchased from Mirus Bio (Madison, WI). A Microvette-CB300-LH was from Fisher Scientific (Pittsburgh, PA). Thermocouple Meter was from Kent Scientific Corp (Torrington, CT). The insulin ELISA kit was obtained from Mercodia Developing Diagnostics (Winston Salem, NC). A TUREtrack glucometer and test strips were purchased from Nipro Diagnostics, Inc. (Fort Lauderdale, FL). The Oil Red O staining solution was obtained from Electron Microscopy Science (Hatfield, PA). The high-fidelity DNA polymerase was purchased from NEB (Ipswich, MA). The TRIZOL reagent and the SuperScript^®^ III First-Strand Synthesis System were purchased from Life Technologies (Grand Island, NY). The RNeasy Lipid Tissue Mini Kit was from Qiagen (Valencia, CA). PerfeCTa^®^ SYBR^®^ Green FastMix was acquired from Quanta BioSciences (Gaithersburg, MD).

### Plasmid construction

A coding region of mouse *Nrg4* (NCBI GenBank, NM_032002) was cloned from complementary DNA sequences of C57BL/6 mouse liver and inserted into a pLIVE vector at the *NheI* and *XhoI* cutting sites. The insertion in the new plasmid (pLIVE-NRG4) was confirmed by DNA sequencing. The control plasmid pLIVE-SEAP contains the same backbone and a secreted alkaline phosphatase gene. Both pLIVE-NRG4 and pLIVE-SEAP plasmids were purified by CsCl–ethidium bromide density-gradient ultracentrifugation and kept in saline (0.9% sodium chloride). Purity of the plasmids was verified by absorbency ratio at 260 and 280 nm and by 1% agarose gel electrophoresis.

### Animals and treatment

All procedures performed on animals were approved by the Institutional Animal Care and Use Committee at the University of Georgia, Athens, Georgia and the methods were carried out in accordance with the approved protocol #A2014-07-008-Y1-A0. For the prevention study, C57BL/6 mice (male, 8-week old) were housed under standard conditions with a 12 h light-dark cycle and fed an HFD for a total of 9 weeks. For the treatment study, mice were first fed an HFD for 25 weeks, reaching a body weight of 56 grams on average. Gene transfer was performed on the obese mice and the treated animals were continued on an HFD for additional 3 weeks. Hydrodynamic gene delivery was performed according to a pre-established procedure^10^,^41^,^42^. Briefly, an appropriate volume of saline solution (equivalent to 8% lean mass) containing 20 μg pLIVE-NRG4 or pLIVE-SEAP was injected through the tail vein within 5–8 sec. The body weight of each mouse was measured on an electronic balance, and body composition was analyzed using an EchoMRI-100^TM^ (Echo Medical Systems, Houston, TX). Food intake per mouse was calculated based on the amount consumed divided by time and the number of mice per cage. Blood was collected at the desired time using a Microvette-CB300-LH. Rectal temperature of the mice was measured at the desired time using a specially designed Thermocouple Meter.

### Histochemical analysis

After the mice were euthanized, the liver, EWAT, SubWAT, and BAT were collected and fixed in 10% formalin. Paraffined tissues were sectioned at a thickness of 6 μm and stained with H&E solution. For Oil Red O staining of liver sections, freshly collected liver samples were frozen in liquid nitrogen. Frozen sections (8 μm) were made and stained with 0.2% Oil Red O in 60% of isopropanol for 20 min and washed three times with phosphate buffered saline. A microscopic examination was performed and photographs were taken under a regular light microscope.

### Glucose tolerance test (GTT) and insulin tolerance test (ITT)

The analyses were performed on week nine post gene transfer in the prevention study and on week three after gene transfer to obese mice in the treatment study. For GTT, mice were fasted for 6 h and injected intraperitoneally with glucose at 1.5 g/kg body weight. Blood samples were collected from the tail vein and glucose levels were measured using a glucometer. For ITT, mice were fasted for 4 h and intraperitoneally injected with insulin (0.75 U/kg, Eli Lilly Indianapolis, IN). Blood samples were collected from the tail vein and glucose levels were measured using a glucometer.

### Gene expression analysis by real time PCR

Total RNA was isolated from the liver, pancreas, BAT, and EWAT using the TRIZOL reagent or an RNeasy kit. Two micrograms of total RNA were used for the first strand cDNA synthesis, as recommended by the manufacturer. Real time PCR was performed, using SYBR Green as an indicator, employing an ABI StepOne Plus Real Time PCR system. PCR was carried out for 40 cycles at 95 °C for 15 s and 60 °C for 1 min. Fluorescence was read during the reaction, allowing a continuous monitoring of the amount of PCR product. The data were normalized using *Gapdh* mRNA as an internal control. The primer sequences employed are summarized in Supplemental Table S1.

## Statistical analysis

A statistical analysis was performed using the Student’s *t* test. All data are reported as mean ± standard deviation (SD) with statistical significance set at *P* < 0.05.

## Additional Information

**How to cite this article**: Ma, Y. *et al*. Preventing High Fat Diet-induced Obesity and Improving Insulin Sensitivity through Neuregulin 4 Gene Transfer. *Sci. Rep.* **6**, 26242; doi: 10.1038/srep26242 (2016).

## Supplementary Material

Supplementary Information
