# Supplementary material for: Preventing High Fat Diet-induced Obesity and Improving Insulin Sensitivity through Neuregulin 4 Gene Transfer
Source: Sci Rep. 2016 May 17;6:26242. doi: 10.1038/srep26242 (PMC4869101; doi:10.1038/srep26242)
Supplement: Supplementary Information [file srep26242-s1.pdf]

Preventing High Fat Diet-induced Obesity and Improving Insulin Sensitivity through Neuregulin 4 Gene Transfer

Yongjie Ma[1], Mingming Gao[1] and Dexi Liu[1]*

Supplementary Table S1

Supplementary Figure S1 and Figure Legend

Supplementary Figure S2 and Figure Legend

Supplementary Figure S3 and Figure Legend

Supplementary Figure S4 and Figure Legend

Supplemental Data

Table S1 Primer sets for real time PCR analysis of gene expression

Name	Forward sequence	Reverse sequence
F4/80	CCCCAGTGTCCTTACAGAGTG	GTGCCAGAGTGGATGTCT
Cd68	CCATCCTTCACGATGACACCT	GGCAGGGTTATGAGTGACAGTT
Cd11b	ATGGACGCTGATGGCAATACC	TCCCCATTCACGTCTCCCA
Cd11c	ACGTCAGTACAAGGAGATGTTGGA	ATCCTATTGCAGAATGCTTCTTTACC
Mcp1	ACTGAAGCCAGCTCTCTCTTCCTC	TTCCTTCTTGGGGTCAGCACAGAC
Tnfα	CCCTCACACTCAGATCATCTTCT	GCTACGACGTGGGCTACAG
Atgl	CAACGCCACTCACATCTACGG	TCACCAGGTTGAAGGAGGGAT
Adiponectin	AGCCGCTTATATGTATCGCTCA	TGCCGTCATAATGATTCTGTTGG
Nrg4	ATGCCAACAGATCACGAGC	TCTTCAGTGTTCTCTGTGGCTG
Ucp1	AGGCTTCCAGTACCATTAGGT	CTGAGTGAGGCAAAGCTGATTT
Ucp3	ATGAGTTTTGCCTCCATTCG	GGCGTATCATGGCTTGAAAT
Pgc1α	GAAGTGGTGTAGCGACCAATC	AATGAGGGCAATCCGTCTTCA
Dio2	AATTATGCCTCGGAGAAGACCG	GGCAGTTGCCTAGTGAAAGGT
Cidea	ATCACAACTGGCCTGGTTACG	TACTACCCGGTGTCCATTTCT
Insulin1	CACTTCCTACCCCTGCTGG	ACCACAAAGATGCTGTTTGACA
Insulin2	GCTTCTTCTACACACCCATGTC	AGCACTGATCTACAATGCCAC
Pepck	AAGCATTCAACGCCAGGTTC	GGGCGAGTCTGTCAGTTCAAT
G6pase	CGACTCGCTATCTCCAAGTGA	GTTGAACCAGTCTCCGACCA
Srebp-1c	CCCTGTGTGTACTGGCCTTT	TTGCGATGTCTCCAGAAGTG
Acc-1	GCCTCTTCCTGACAAACGAG	TGACTGCCGAAACATCTCTG
Fas	AGAGATCCCGAGACGCTTCT	GCCTGGTAGGCATTCTGTAGT
Scd-1	TTCTTACACGACCACCACCA	CCGAAGAGGCAGGTGTAGAG
Cyp7a1	AACGGGTTGATTCCATACCTGG	GTGGACATATTTCCCCATCAGTT
Hmgcr	CTTGTGGAATGCCTTGTGATTG	AGCCGAAGCAGCACATGAT
Abca1	AAAACCGCAGACATCCTTCAG	CATACCGAAACTCGTTCACCC
Pparγ1	TTTTCCGAAGAACCATCCGATT	ATGGCATTGTGAGACATCCCC
Pparγ2	TCGCTGATGCACTGCCTATG	GAGAGGTCCACAGAGCTGATT
Cd36	CCTTAAAGGAATCCCCGTGT	TGCATTTGCCAATGTCTAGC
Fabp4	AAGGTGAAGAGCATCATAACCC	TCACGCCTTTCATAACACATTCC
Mgat1	TGGTGCCAGTTTGGTTCCAG	TGCTCTGAGGTCGGGTTCA
Pparα	TGTCGAATATGTGGGGACAA	AATCTTGCAGCTCCGATCAC
Cpt1a	CTCCGCCTGAGCCATGAAG	CACCAGTGATGATGCCATTCT
Cpt1b	GGTCTCTTCTTCAAGGTCTG	CGAGGATTCTCTGGAACTGC
Gapdh	AGGTCGGTGTGAACGGATTTG	TGTAGACCATGTAGTTGAGGTCA

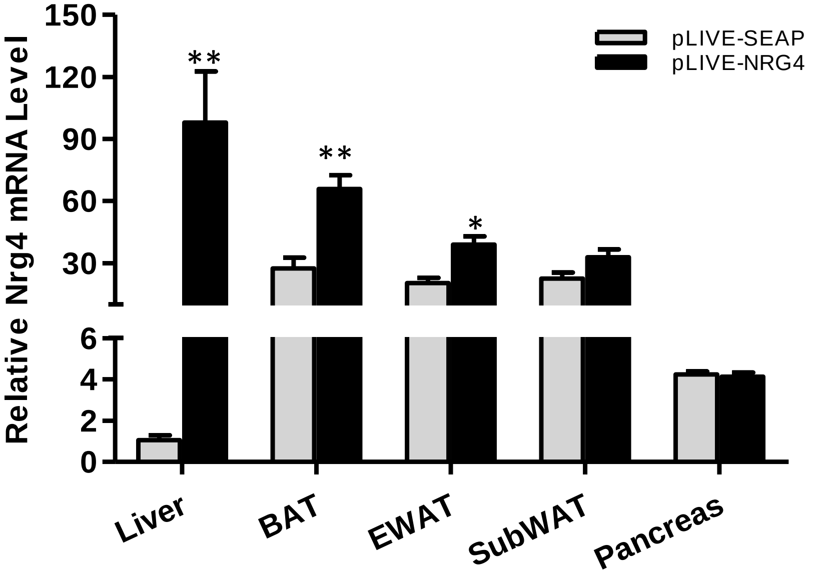

Figure S1 *Nrg4* gene expression in different tissues. Eight-week-old C57BL/6 male mice were hydrodynamically injected via tail vein of 20 μg of pLIVE-NRG4 or pLIVE-SEAP control pasmid DNA and fed a HFD for 9 weeks. At the end of experiment, total RNA was extracted from liver, EWAT, SubWAT, BAT, pancreatic tissues, and the relative mRNA levels of *Nrg4* gene were determined by real-time or regular PCR. *P<0.05, **P<0.01 compared to that of control animals injected with pLIVE-SEAP *(n=5).*

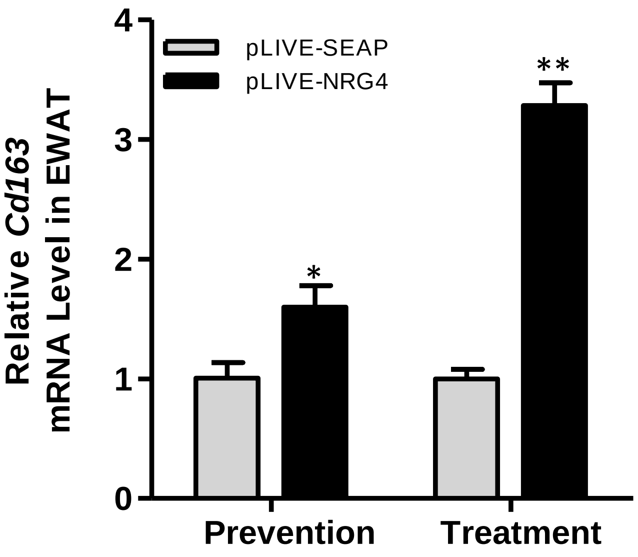

Figure S2 ***Nrg4*** **gene transfer increased M2 macrophage marker** ***Cd163*** **in EWAT.**

At the end of experiment, total RNA was extracted from EWAT and the relative mRNA levels of *Cd163* gene were determined by real-time. *P<0.05, **P<0.01 compared to that of control animals injected with pLIVE-SEAP *(n=5).*

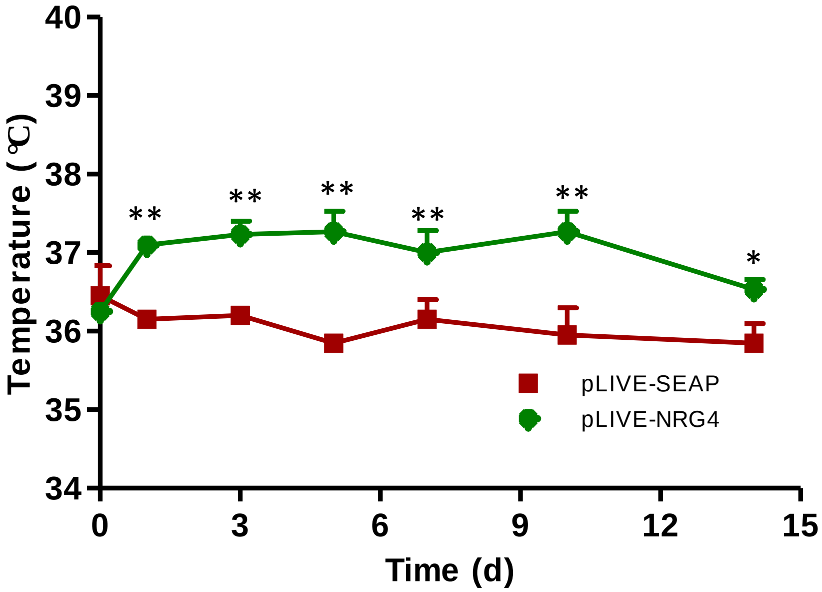

Figure S3 *Nrg4* gene transfer increased mice body temperature

After C57BL/6 male mice were hydrodynamically injected via tail vein of 20 μg of pLIVE-NRG4 or pLIVE-SEAP control plasmid DNA. Rectal temperature was measured at desirable time after gene delivery. *P<0.05, **P<0.01 compared to pLIVE-SEAP group. (n=5)

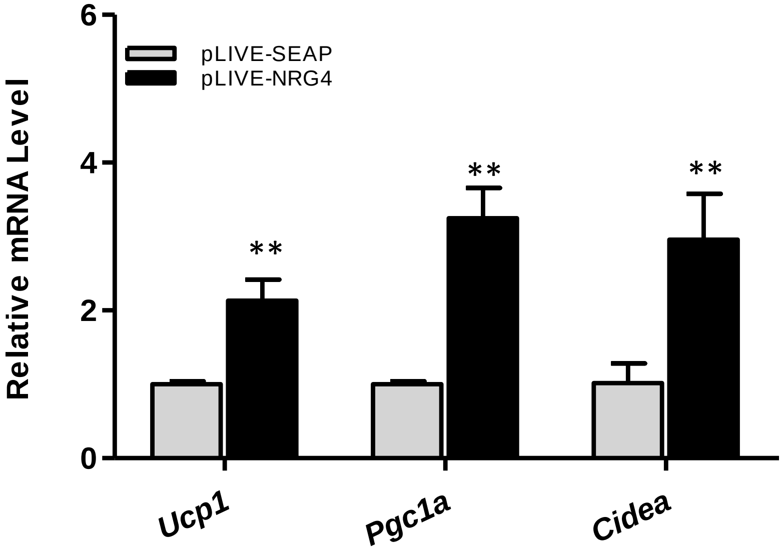

Figure S4 ***Nrg4*** **gene transfer increased browing maker genes in inguinal WAT**.

At the end of experiment, total RNA was extracted from inguinal WAT and the relative mRNA levels of thermogenic genes expression were determined by real-time. **P<0.01 compared to that of control animals injected with pLIVE-SEAP *(n=5)*.
